# A randomized, controlled study to evaluate the efficacy of intra-articular, autologous adipose tissue injections for the treatment of mild-to-moderate knee osteoarthritis compared to hyaluronic acid: a study protocol

**DOI:** 10.1186/s12891-018-2300-7

**Published:** 2018-10-24

**Authors:** Ian A. Jones, Melissa Wilson, Ryan Togashi, Bo Han, Austin K. Mircheff, C. Thomas Vangsness JR

**Affiliations:** 1 Department of Orthopaedic Surgery, Keck School of Medicine of USC, HCT 1520 San Pablo Street, suite 2000, Los Angeles, CA 90033 USA; 2 Department of Preventive Medicine, Keck School of Medicine of USC, 2001 Soto Street, SSB1 318A, Los Angeles, CA 90033 USA; 3 Departments of Surgery and Biomedical Engineering, Keck School of Medicine of USC, 1333 San Pablo St. BMT-302, Los Angeles, CA 90033 USA; 4 Department of Physiology & Neuroscience, Keck School of Medicine of USC, 1333 San Pablo St. BMT B-11A, Los Angeles, CA 90033 USA

**Keywords:** Lipogems, Stem cell, Knee, Osteoarthritis, Intra-articular injection, Adipose tissue, Fat MSC, ADSC

## Abstract

**Background:**

Osteoarthritis (OA) is a highly debilitating joint disease that causes progressive, irreversible damage to articular cartilage. OA takes a massive toll on society that has grown in recent decades, but no therapy has been shown to halt or reverse the progression of the disease. The critical need for better treatments and increased interest cellular therapies has spawned a new generation of “minimally manipulated” cell treatments. Autologous adipose tissue injections are among the most controversial of these new treatments. Despite a lack of clinical evidence, adipose tissue injections are often marketed as “stem cell” injections with wide-ranging regenerative benefits. The purpose of this study is to estimate the effect size of the treatment by comparing the efficacy of autologous fat to hyaluronic acid (HA). As a secondary aim, we will test for preliminary evidence of efficacy of autologous fat vs. HA.

**Methods:**

This is a prospective, single-center, parallel-group, randomized, controlled trial. Participants (*n* = 54) will receive either a single intra-articular, ultrasound-guided injection of autologous adipose tissue or a single intra-articular, ultrasound-guided injection of HA (1:1 ratio). Outcome data will be obtained at baseline, week-6 and month-6. The Western Ontario and McMaster Universities Osteoarthritis Index (WOMAC) pain domain (WOMAC-A) will be used as the primary outcome measure. Secondary clinical outcome measures include WOMAC (full), clinical anchors (pain, function, and stiffness), and the 29-point Patient-Reported Outcomes Measurement Information System (PROMIS®) profile. We will also take synovial fluid samples and assess sway velocity using a force plate, as well as analyze excess/discard adipose tissue to gain a better understanding of how intra-articular adipose tissue injections influence the biochemical environment of the joint.

**Discussion:**

Given the widespread use of intra-articular fat injections in the United States, it is critical that randomized, controlled human studies evaluating efficacy and biological activity be performed. This study is the first step in addressing this unmet need, but it is not without limitations. The most notable limitations of this study are its small size and lack of blinding, which predisposes the study to both investigator and participant bias.

**Trial registration:**

NCT03242707 // HS-17-00365 // Registration Date (First Posted): August 8, 2018.

**Electronic supplementary material:**

The online version of this article (10.1186/s12891-018-2300-7) contains supplementary material, which is available to authorized users.

## Background

### Introduction

Osteoarthritis (OA) is a debilitating “whole joint disease” that causes progressive, irreversible damage to articular cartilage that results in debilitating joint pain and stiffness [1]. OA places a massive financial burden ln society [2] that has grown in recent decades [3–5]. There are no approved disease modification therapies for OA, and the only non-invasive pharmacologic therapies recommended for OA symptom management are analgesics and non-steroidal anti-inflammatory drugs (NSAIDs) [6, 7]. Intra-articular therapies, including corticosteroids and hyaluronic acid (HA) are frequently used to treat knee OA, but both treatments have limitations. The American Academy of Orthopaedic Surgeons (AAOS) determined that there was inconclusive evidence to support the use of intra-articular corticosteroids of OA [8] and several recent studies have suggested that corticosteroids may have detrimental catabolic effects on cartilage [9–11]. While generally assumed to be safe, the AAOS concluded that the apparent effectiveness of HA compared to saline placebo is clinically negligible, and strongly recommended against its use [12]. The recommendation has been met by push back [12, 13], and meta-analysis published since have both confirmed [14] and contradicted [15] the appropriateness of the AAOS recommendation. Nevertheless, even if assumed to be effective, HA provides (at best) modest, short-term, symptomatic relief compared to placebo control.

OA is frequently referred to as a “wear and tear” disease. However, OA pathophysiology is multifactorial, and complex interactions between genetic, metabolic, biochemical, and biomechanical factors are also likely to play an important role in the progression of the disease [16, 17]. In recent years, studies have suggested that inflammatory mechanisms contribute to OA pathogenesis, particularly synovitis, which has been correlated with the structural progression of OA, cartilage degeneration and osteophyte formation [18]. Indeed, an emerging body of evidence indicates that OA is a whole-organ disease that involves the production of cytokines by a variety of different tissues [1, 19] and a number of cytokines have been implicated in OA pathogenesis [20–24].

There is considerable interest in exploiting the anti-inflammatory activities of cellular treatments to treat OA [25]; however, adipose tissue-derived cell therapies require enzymatic processing and isolation techniques that may impact safety and efficacy [26]. These concerns have prompted the United States Food and Drug Administration (US FDA) to regulate autologous cell therapies that have been cultured and/or enzymatically processed as drugs. The promise of cellular therapies, particularly those containing so called mesenchymal “stem” or stromal cells (collectively referred to as MSCs), combined with regulatory barriers and the critical need for new therapies, has spawned a new generation of “minimally manipulated” cell treatments for OA. Among these new “minimally manipulated” cell treatments are autologous adipose tissue injections, which are often marketed as “stem” cell injections and are currently available at orthopaedic clinics across the United States [27].

In the US, the rules governing the clinical use of treatments claiming to be “minimally manipulated”, particularly intra-articular adipose tissue injections, are ambiguous. This ambiguity can be attributed to special regulatory exemptions for blood-derived cell products [28], as well as Section 361 of the US Public Health Service Act, which exempts certain human cells, tissues, and cellular and tissue-based products (HCT/Ps) from the regulations that require regulatory submissions for the conduct of clinical trials and marketing [29]. Orthopaedic surgeons administering these treatments claim that they are being administered for reconstruction, repair, and replacement, and that the mechanical processing of the fat does not alter the original relevant characteristics of the transferred tissue [30]. Opponents argue that, despite the presence and importance of fat in the knee joint [31], subcutaneous fat is very different than the adipose tissue of the knee joint, and so the injection of adipose tissue into the intra-articular space should not be considered homologous use. Despite regulatory ambiguity and little evidence demonstrate their effectiveness [32], “minimally manipulated” adipose tissue injections are widely available at clinics throughout the US. To date, there have been no randomized, controlled clinical studies performed to evaluate the efficacy intra-articular adipose tissue injections for the treatment of OA.

### Basic science for clinical study aspects

The principle difference between cell therapies and “minimally manipulated” fat injections is that fat injections are not enzymatically digested. As a result, autologous fat treatments contain both autologous cells, including MSCs [33], and extra-cellular matrix (ECM). Therapies containing ECM are thought to have the following advantages over treatments prepared using enzymatic digestion: (1) preservation of the stromal vascular niche, which allows time-release of the regenerative factors [34]; (2) release of bioactive molecules by exosomes, which have been demonstrated to be significantly greater in mechanically processed fat than enzymatically processed fat [35]; and (3) maintenance of the structural and morphologic unit, which is thought to increase efficacy by making the cells more resilient to the harsh, inflamed conditions in the recipient environment [36]. Enzymatic digestion, which is used to remove the cells from ECM, may remove critical elements involved in tissue repair and may damage the cells, affecting their function and viability. In vitro studies have suggested that cell-containing lipoaspirate obtained without enzymatic digestion may act as a scaffold when administered via intra-articular injection, allowing for the formation of fibrous tissue that provides mechanical support [37]. While non-enzymatic processing produces a lower progenitor cell yield [38, 39], the preserved ECM and growth factors are thought to contribute to the treatments’ overall therapeutic effectiveness.

Preclinical [37] and clinical [40, 41] studies investigating the use of “minimally manipulated” adipose tissue injections have demonstrated preliminary safety, but a number of questions remain unanswered. While ECM has been shown to be beneficial for tissue repair [42] and is commonly used to as a bridging material between new and old tissues [43], these advantages are less applicable to intra-articular fat injections because there are no tissue fragments for the ECM to bridge [44] and because agents injected into the joint tend to be quickly cleared from the body [45]. Most importantly, ECM itself has the potential to produce inflammatory signals [46]. In fact, collagen fragments are known to induce arthritis in animals [47].

### Feasibility justification for clinical aspects of the study

Adipose tissue will be obtained and administered in a single point-of-care procedure. The rapid processing (approx. 20 min) within a closed system reduces the possibility of contamination. However, this also means that the specific composition of the adipose tissue therapy cannot be determined prior to injection. As a result, there will be inherent, unavoidable variability between the treatments that participants receive. In order to help minimize this variability, each patient will receive exactly 6 ml of processed adipose tissue. The decision to inject 6 ml is based on anatomic considerations and the principal investigator’s clinical experience. A 6 ml injection is conservative, as the synovial fluid volume has been estimated to be 6.7 +/− 2.3 ml [48], with the average volume of the knee joint itself being 131 ± 53 ml [49]. The 6 ml “dose” is also in line with published case studies, where fat processed with the Lipogems® device has been injected into the knee [40]. Lastly, a 6 ml volume improves comparability because the comparator treatment is also a 6 ml intra-articular injection.

The entire fat transfer procedure will be completed in under an hour. Adipose tissue will be harvested from the abdomen using lipoaspiration, which does not require general anesthesia [50]. The tissue will be mechanically broken down using the Lipogems® device. While a number of other similar fat processing systems are available [51], the differences between the products produced by different systems and their implications for osteoarthritis are unclear [32].

Our study will use Hyaluronic acid (HA) as an active control. In accordance with the requirements for the administrations for HA, only patients that fail to respond to the standard conservative treatment options (exercise, analgesics, NSAIDS [7]) will be recruited. The use of HA as an active control for autologous cell-based therapies is well-established [52, 53]. We have chosen to use Synvic One® because it has been included in more than 300 publications and has been used to treat more than 13 million knees worldwide. Synvic One® is also an ideal comparator treatment for this study because it has been shown to have an effect that last for up to 6 months [54].

### Study overview

This is a prospective, single-center, parallel-group, randomized, controlled study. The aim of this study is to estimate statistical power and gather preliminary efficacy data. Qualified participants will receive either a single, intra-articular injection of autologous adipose tissue or a single, intra-articular injection of HA. We hypothesize that participants treated with autologous adipose tissue will show a greater improvement in pain (as determined using the pain items of the Western Ontario and McMaster Universities Osteoarthritis Index, or WOMAC-A) than participants treated with HA. WOMAC (full questionnaire), the 29-point Patient-Reported Outcomes Measurement Information System (PROMIS®) profile, and clinical anchors will be used as secondary clinical outcome measures. Synovial fluid samples and excess adipose tissue (treatment group) will also be analyzed to gain a better understanding of how adipose tissue affects the biochemical environment of the joint.

## Methods

### Participants, interventions, and outcomes

**Fig. 1:**
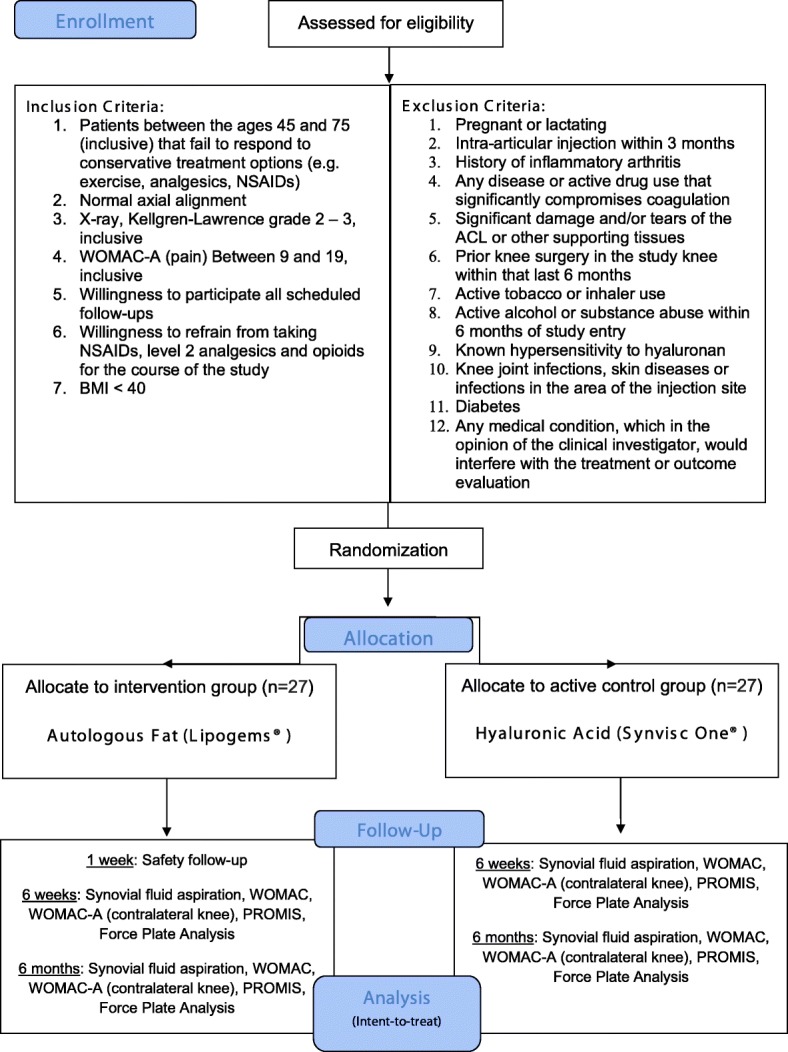
Study design flow chart

All study-related activities will be performed at the USC Department of Orthopaedic Surgery Outpatient Clinic, including subject identification, screening, treatment, and outcome assessments. Patients between the ages of 45 and 75 with Kellgren-Lawrence grades 2–3 OA (inclusive) that are interested in participating in the study will be screened for eligibility by the principal investigator using pre-defined criteria (Fig. 1). Those who meet inclusion/exclusion criteria and decide to participate will sign an informed consent before randomization (see Assignment of Interventions). Because the fat treatment requires the removal of adipose tissue from the abdomen, participants will not be blinded to their assigned group.

Male and/or female patients will be randomized to either the HA (Synvic One®) treatment group (*n* = 27) or autologous fat (Lipogems®) treatment group (n = 27). To ensure enrollment goals are met, the investigator will draw from a waitlist of current patients that have expressed interest in participating in clinical trials. We also have IRB-approved flyers/brochures that will be shared with patients and nearby orthopaedic clinics (Additional file 1). Additionally, to improve adherence to the intervention protocol, participant who are allocated to the control group that complete all scheduled follow-up visits will be offered the adipose tissue treatment at the end of their final visit at no cost.

Since our primary aim is to estimate the effect size for future well-powered studies, we estimate the precision around which we can measure the effect. Based on a prior study using similar methods, but comparing WOMAC-A scores among patients receiving placebo to Hylan G-F (a joint lubricant) [54], we assumed standard deviations in the control and treatment groups of 0.66 and 0.67, respectively. With a sample size of 54 (27 per group), we will be able to estimate a two-sided 95% confidence interval within ±0.36 distance from the mean difference. For our secondary aim, we estimated the mean difference we would be able to detect, assuming a similar effect size for HA as was observed for Hylan G-F (μ = − 0.84, σ = 0.67) and found that we would have 80% power to detect a mean difference of − 1.4 in the autologous adipose group with a two-sided Type I error rate of 0.05.

Power was calculated using Pass 14 (Kaysville, UT).

At the treatment visit (day 0), patients will be randomly assigned to receive either a single intra-articular, ultrasound-guided injection autologous adipose tissue (6 ml) or a single intra-articular, ultrasound-guided injection of hyaluronic acid (6 ml). Patients with bilateral OA will be allowed to participate in this study, but only their more painful eligible knee will be treated (as determined by WOMAC-A). To control for possible responder differences between bilateral and unilateral symptomatic patients [55, 56], WOMAC-A for the contralateral knee will be obtained at baseline, 6 weeks, and 6 months (Table 1). If a participant has bilateral OA of equal pain intensity in both knees, the treatment knee will be selected randomly.

**Table 1:** Study Calendar

Timepoint**	Study period				
	Screening	Allocation	Follow-up visits		Close-out
	Day − 21 to 0	day 0	Safety follow-up visit Day 2–7 (inclusive)	Week 6 ± 7 days	Month 6 ± 30 days
Enrolment:					
Eligibility screen	X				
Informed consent	X				
*Focused physical exam*		X	X	X	X
Randomization / Allocation		X			
Interventions:					
*Autologous Adipose Tissue Injection*		X			
*Hyaluronic Acid Injection*		X			
Assessments:					
WOMAC	X	X		X	X
WOMAC-A (contralateral knee)	X	X		X	X
PROMIS-29		X		X	X
Force Plate Analysis (bilateral)		X		X	X
Clinical anchors		X		X	X
Synovial fluid aspiration		X		X	X

The *eligibility screen* includes a detailed physical exam/medical history and pregnancy testing (if applicable). The *focused physical exam* includes Height/weight/vitals, concomitant medications, knee exam, and wound evaluation (if applicable). Only patients that receive the fat treatment will be asked to come in for the safety follow-up. All assessments pertain only to the study knee, unless otherwise indicated

#### Lipoaspiration procedure

For patients receiving the adipose tissue injection, fat tissue will be harvested under semi-sterile conditions (sterile draping and gloves, gown, face masks, and head cover). The participant will be positioned supine on an examination bench. Local anesthesia (2% lidocaine) will be applied to the area to be harvested (approx. 8 cm × 20 cm just below the umbilicus) and a small incision (6-8 mm) will be made. Approximately 500 mL of normal saline containing 50 mL of 2% lidocaine and 1amp epinephrine will be infused to reduce bleeding and trauma. Approximately 15 min will be allowed for infiltration. Then, a thin cannula will be inserted through the incision and a controlled back and forth motion will be used to loosen excess fat. Using a 13-gauge cannula connected to a VacLok® syringe, 20 ml to 40 ml of subcutaneous adipose tissue will be harvested. Following the harvest procedure, a pressure dressing will be applied. Participants will be told to remove the dressing 24–48 h after the procedure and return approximately 1 week after receiving the treatment so that the harvest site can be evaluated.

#### Injection procedure

The same injection procedure will be used for both treatments. With the patient in the supine position, the affected knee will be extended, and the femoral condyle will be marked with a surgical marker. The area will be cleaned, and local anesthesia will be applied to the injection site (2 cc, 1% lido). A 21-gauge needle will be inserted into the suprapatellar pouch under ultrasound guidance. Synovial fluid will be aspirated from the knee and autologous fat or HA will be administered through the same needle used to aspirate the synovial fluid. The synovial fluid aspiration portion of the procedure will also be performed at the 6-week and 6-month follow-up visits. A detailed summary of study procedures is provided in Table 1.

The mean change in WOMAC-A score over time will be used as the primary outcome measure. The PROMIS-29 profile, clinical anchors and WOMAC will be used as secondary clinical outcome measures. WOMAC is multidimensional, self-administered health status instrument for patients with OA of the hip and/or knee and been shown to fulfill conventional criteria for content and construct validity, reliability, responsiveness and relative efficiency [57]. The PROMIS-29 questionnaire is set of person-centered measures that encompasses domains that OA is likely to affect, including functional limitations, pain interference/intensity, ability to fulfill desired social roles, anxiety/depression, sleep disturbance and fatigue [58]. PROMIS-29 has been shown to correlate with scales measuring similar constructs for patients with OA [59] and is frequently used for research purposes [58]. Three clinical anchors (pain, function and stiffness; 7-point Leichhardt scale) will be used to determine the patient’s global impression of change, as described previously [60, 61].

We will assess participants using a proprietary system that includes a force plate (1000 Hz (9260AA6, Kistler Instruments, Winterthur, Switzerland), dedicated computer, and software (SpartaMARS, Sparta Performance Science, Menlo Park, USA). Participants will be assessed at baseline, 6 weeks and 6 months. The non-invasive standing assessment will take approximately 3 min to complete. Assessments will be performed after clinical outcome data has been obtained, but before synovial fluid has been aspirated. Participants will be instructed to stand on the scale with their hands on their hips and eyes closed to establish baseline force. The test consists of four, 20 s balance trials on each leg. A 10 s rest period will be provided between trials, and trials will be conducted on alternating legs. Displacement and sway velocity will be measured as described previously [62]. The results of the force plate assessment will be associated with clinical outcome data and synovial fluid biomarker profiles. Results of the force plate sub-set analysis will be reported separately.

Excess adipose tissue will be transferred to sterilized vials filled with phosphate buffered saline. Adipose tissue samples will be kept at 4 °C. Samples will be divided into two parts in the biosafety hood using sterile scalpel. One part of each sample will be processed for cell culture, and another part will be cryopreserved. For cell culture, samples will be transferred to a tube containing RPMI serum free medium, supplemented with 1% penicillin-streptomycin-fungizone. Tissue samples will be washed 3 times with the same medium, then finely minced for cell isolation using a standard collagenase digestion protocol.

Synovial fluid samples will be transferred to EDTA tubes, gently inverted 8–10 times, and then immediately transported to the lab on wet ice in accordance with university policies. Samples will be centrifuged within 1 h at 1500 x g for 10 min at 4 °C. The supernatant will then be aliquoted to cryovials containing protease inhibitor and stored at -80C until use.

Synovial fluid samples and excess adipose tissue will be analyzed to characterize the treatment and gain a better understanding of how adipose tissue affects the biochemical environment of the knee. To better monitor the disease progression and treatment efficacy, chemokines, cytokines, growth factors, and matrix metalloproteinases in the synovial fluid will be analyzed, including IL-1β, IL-6, IL-8, TNFα, C-terminal telopeptides of Type I collagen, and C-telopeptide of Type II collagen. The results will be made available for principle component analysis, which will be used to determine if pre-treatment synovial fluid inflammatory mediator and selected OA biomarker profiles that define different OA phenotypes predict the responses to autologous adipose tissue injections. The pro- and anti-inflammatory capabilities and cell viability of the autologous fat product will be evaluated and may also be compared to unprocessed preparations.

The use of NSAIDs may negatively impact healing [32, 63] and their prolonged half-life in synovial fluid allows them to accumulate in the joint [64], which is likely to impact synovial fluid biomarker profiles. To ensure that clinical outcomes, tissue healing and the biochemical profile of the synovial fluid are not influenced by the use of NSAIDs, participants will be asked not to take NSAIDs for at least 1 week prior to treatment and throughout the duration of the study.

Patients will be instructed to contact the study doctor before taking analgesic medications. Acetaminophen (250-500 mg) will be recommended if appropriate. The total number of days each patient takes acetaminophen will be logged and reported with published results. However, extended acetaminophen used will not be used as grounds for termination, as it has it is generally thought to have a clinically negligible effect on OA-related knee pain [65–67].

### Assignment of interventions

A random number sequence was generated using the Stata 14.0 uniform distribution. Blocks of four were used to ensure equal numbers of subjects in each group. To carry out the randomization of study subjects, we will utilize sequentially numbered, opaque, sealed envelopes. The statistician will prepare the envelopes for the Principal Investigator in order to maintain the concealment of the sequence prior to randomization. Upon enrolment in the trial, the next sequentially numbered envelope will be selected by the investigator and the subject will be offered the assigned treatment.

### Data collection, management, and analysis

Demographics and survey data will be stored using a REDcap database created specifically for the purpose of this study. REDcap has several security features, including off-site backups, an audit trail, secure logins, de-identified data exports, and built-in filtering methods to ensure data quality. Additionally, survey answers (clinical outcome data) will be directly entered into the database by the participants via iPad before they interact with the examining physician. Documentation not included in the quantitative analysis of clinical outcomes will be recorded using traditional source documents (Additional files 2 and 3). These documents will be kept in the participant’s binder and locked in a secure location. All data collection will take place in the clinic.

Only those who require access to the database will be given access. Coding of the variables will be done within the database. A completed data file will be stored by the statistician in the shared folder for the Clinical Translational Science Institute Biostatistics core at USC and will be backed up regularly. The statistician’s access to the database will be limited to de-identified data. Data quality will be assured by range checks for unusual or impossible data values. Data management will include a thorough data cleaning (checking ranges, evaluating distributions) and deriving of variables needed for analysis.

All protocol deviations will be recorded. The same outcome data will be obtained from all participants, regardless of protocol adherence. In order to promote retention and compliance, participants that receive the HA treatment and complete all schedule follow-up visits will be offered the fat treatment at the 6-month (final) follow-up visit at no charge. Imputation will be used if loss to follow-up is uncommon.

Preliminary analyses will include descriptive statistics of basic demographic and clinical characteristics to assess the degree to which randomization was effective in balancing treatment groups. If any factors are found to have clinically significant differences, they will be considered and evaluated as possible confounders. The analysis will be by intent-to-treat. That is, all subjects will be analyzed according to randomization status, regardless of actual treatment received, compliance with therapy, or adherence to the study protocol. We will compare the outcome, difference in WOMAC-A score from baseline to 6 months, between the treated and untreated groups using generalized linear models (GLM) with a Gaussian family specified, assuming the data are normally distributed. If the data are not normally distributed, we will specify a more appropriate distribution based on an evaluation of the data via histogram. We will also use GLM to examine the relationship between the treatment and outcome at each time point at which the WOMAC was measured. Last, we will model the effect across all time points using a multi-level mixed effects model to account for repeated measures. Confounders will be defined as those variables that were found to differ between groups either at baseline or post-randomization and which, when added to the model, alter the effect estimate by > 15%. Potential a priori confounders include BMI, Bilateral vs contralateral OA, gender, ethnicity, and race. Potential interactions between treatment and visit will be evaluated. A *p*-value of <.05 will be considered statistically significant. The 29-point PROMIS® questionnaire will be evaluated using GLM at each measured time point and across time points using a multi-level mixed effects model, as described above. Missing data, if ≤5%, will be imputed using multiple imputation methods. Any covariate missing > 5% will not be included in the analysis.

A per-protocol analysis will also be conducted including only those participants who were protocol-adherent. Protocol adherence is defined as: (1) Receiving the assigned study treatment and (2) completing all follow-up visits. Lastly, sensitivity analyses will be conducted to assess the impact of using multiple imputation methods, if such methods are employed in the analysis.

### Monitoring

This is a small-scale, single-site, investigator-initiated clinical trial, so a data monitoring committee will not be utilized. The principle investigator will regularly review study data for the occurrence of adverse events (AEs), including moderate to severe effusion, synovitis, local infection, systemic infection, and toxicities. The study will be suspended if more than 1 out of the first 10 patients enrolled in the study group experiences a significant adverse event (SAEs) attributable to the investigational treatment. SAEs are defined (per US FDA) as those that are life threatening, require hospitalization, or result in death, disability or long-term damage. All adverse events (AEs and SAEs) will be recorded in study source documents and will be reported to the University IRB in accordance with federal, state and institutional requirements. Additionally, all AEs and SAEs will be reported with published results.

### Ethics and dissemination of information

Ethics approval was received from the University of Southern California Health Science IRB (10/9/2017), USC Institutional Biosafety Committee (May 2017) and USC Radiation Safety Committee (September 2017). Continuing reviews and all protocol modifications will be submitted to the USC Health Science IRB through the University iStar System. Because the IRB has determined that this study involves ‘greater than minimal risk’, only the Principal Investigator will be allowed to obtain informed consent. All participants provided written informed consent to participate. Participants will be given a copy of the consent to take home, and an informed consent comprehension assessment will be performed before randomization.

To protect participant confidentiality, samples will not be labeled with information that can be used to directly identify participants. Samples will be labeled with subject identification numbers and only the investigator and IRB-approved study personnel will have access to information linking subject identification numbers to identifiable information. The link between study participants and their study ID numbers will be destroyed (shredded/purged) in accordance with federal regulations when study activities are complete. The final trial dataset will not contain identifiable data and will be available to all investigators listed in the IRB protocol.

There are no additional provisions for ancillary or post-trial care, or for compensation to those who suffer harm from trial participation. However, participants will be informed prior to enrollment that they are responsible for unscheduled visits/interventions and that they are not giving up any legal rights by agreeing to participate in the study (Additional file 4). Once study activities have been completed, results will be published and posted to clinicaltrials.gov.

Personal information will be collected using REDcap, which maintains a secure login and will only be accessible to appropriately delegated study staff, as indicated on the IRB protocol. De-identified data exports will be performed so that statisticians and collaborators may receive data sets without knowledge of personal health information. A de-identified dataset will be stored with the statistician indefinitely. The results of the study, whether positive or negative, will be published. An abstract based on the results may be submitted to an orthopaedics meeting prior to publication. Should the results prove impactful, we may contact the Keck Media Relations Team at USC to assist in developing a press release.

## Discussion

In view of the already widespread use of intra-articular fat injections in the United States, it is critical that randomized, controlled studies aimed at evaluating the clinical efficacy of intra-articular autologous adipose tissue injections for osteoarthritis be performed [68]. This study is the first step towards addressing this unmet need, but it is not without limitations. The most notable limitation of this study is its small size, which is fixed due to funding constraints. However, in order to consider adopting autologous adipose tissue injections as a new therapy for OA, the clinical impact of the treatment would have to be substantial, as autologous fat injects are more costly, carry additional risks, and are more invasive than HA injections. Thus, detecting a small, statistically significant but clinically negligible difference would not advance the treatment of OA.

Another major limitation is the lack of blinding, which predisposes the study to both investigator and subject bias. However, the trauma associated with the removal of adipose tissue from the abdomen is not justified in patients receiving the control (hyaluronic acid). To minimize investigator bias, which is particularly noteworthy given that the study isn’t multi-site, clinical outcomes will be assessed using self-reported measurement tools and responses will be directly entered into a REDcap database by the participants before they interact with the examining physician. Additionally, other response indictors (synovial fluid analysis, fat characterization) will be blinded for analysis.

The removal of synovial fluid presents minimal risk to patients and may help us better understand how autologous fat injections impact the biological environment of the joint. However, puncturing the joint cavity may have clinically important implications. To mitigate this potential limitation, the joint cavity will only be punctured once at the treatment visit. Additionally, outcome assessments will be performed before removing joint fluid and all synovial aspirations will be performed under ultrasound guidance. The number of successful/failed aspirations in each group will be logged and reported in the final published results. While synovial fluid can be challenging to obtain, prior studies conducted by the investigator and others have demonstrated feasibility [69, 70]. Based on the experience of the investigator, > 50% of aspirations are expected to yield analyzable synovial fluid samples.

This study has several strengths. While imperfect, prospective, randomized controlled trials are the gold standard for evaluating treatment effects because they mitigate selection and information bias, as well as confounding variables. Additionally, this study will use WOMAC, which is generally thought to be the gold standard for assessing patient-relevant treatment effects in osteoarthritis [71, 72]. Moreover, WOMAC has been shown to be more responsive than other outcome measures in short-term, small-scale studies [73]. Finally, we will characterize both the treatment (though analysis of discarded adipose) and the biologic response (though the analysis of synovial fluid), which is critical for understanding the treatment bioactivity [74].

Several aspects of this study were changed based on reviewer recommendations. No patients were recruited prior to the implementation and IRB approval of these changes.

## Additional files

Additional file 1: Recruitment flyer. (PDF 1037 kb)

Additional file 2: **Table S1.** Specialized source documentation for data recording. (DOCX 13 kb)

Additional file 3: **Table S2.** Source document schedule. (DOCX 13 kb)

Additional file 4: ICF document. (PDF 657 kb)

## Abbreviations

AE: Adverse event
ECM: Extra-cellular matrix
GLM: Generalized linear models\
HA: Hyaluronic acid
NSAID: Non-steroidal anti-inflammatory Drug
OA: Osteoarthritis
PROMIS: Patient-Reported Outcomes Measurement Information System
SAE: Severe adverse event
WOMAC: Western Ontario and McMaster Universities Osteoarthritis index
