# Supplementary material for: A randomized, controlled study to evaluate the efficacy of intra-articular, autologous adipose tissue injections for the treatment of mild-to-moderate knee osteoarthritis compared to hyaluronic acid: a study protocol
Source: BMC Musculoskelet Disord. 2018 Oct 24;19:383. doi: 10.1186/s12891-018-2300-7 (PMC6201482; doi:10.1186/s12891-018-2300-7)
Supplement: Supplementary file 1 — Recruitment flyer. (PDF 1037 kb) [file 12891_2018_2300_MOESM1_ESM.pdf]

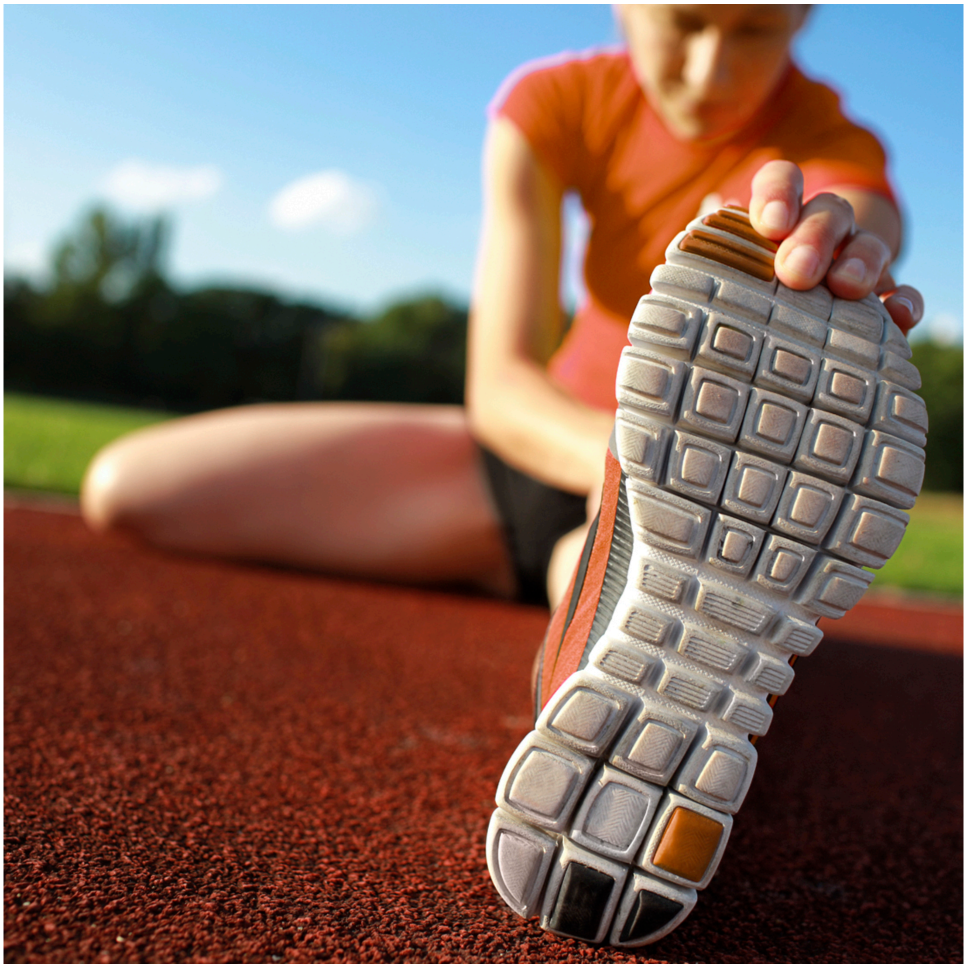

ADIPOSE TISSUE INJECTIONS FOR OSTEOARTHRITIS

A randomized, controlled trial at Keck of USC

Non-operative treatment options for patients with osteoarthritis (OA) are limited. Adipose tissue (or fat) injections are currently offered at many clinics across the country. These treatments are often marketed as "stem cell" or regenerative treatments with wide-ranging benefits. However, very few studies have been conducted. This 6-month study will evaluate the efficacy of adipose tissue injections for patients with mild-to-moderate knee OA compared to hyaluronic acid.

Keck School of Medicine of USC
Department of Orthopaedic Surgery

NOW ENROLLING PATIENTS BETWEEN THE AGES OF 45 AND 75 WITH MILD-TO-MODERATE KNEE OSTEOARTHRITIS

TREATMENT AND FOLLOW-UP VISITS PROVIDED AT NO COST

NO SURGERY REQUIRED

FOR MORE INFORMATION, PLEASE CALL 323-442-6959

C. THOMAS VANGSNESS JR., MD
www.VangsnessMD.com

IRB #: HS-17-00365

Version: 26 APR 2018

NCT # 03242707
