# Supplementary material for: A randomized, controlled study to evaluate the efficacy of intra-articular, autologous adipose tissue injections for the treatment of mild-to-moderate knee osteoarthritis compared to hyaluronic acid: a study protocol
Source: BMC Musculoskelet Disord. 2018 Oct 24;19:383. doi: 10.1186/s12891-018-2300-7 (PMC6201482; doi:10.1186/s12891-018-2300-7)
Supplement: Supplementary file 2 — Table S1. Specialized source documentation for data recording. (DOCX 13 kb) [file 12891_2018_2300_MOESM2_ESM.docx]

**Additional File 3**: Supplementary Table 1: Specialized source documentation for data recording.

| **Document** | **Description** |
| --- | --- |
| Eligibility | Checklist of inclusion/exclusion criteria |
| Informed Consent | Checklist to ensure that a verbal comprehension assessment has been administered and all documentation has been signed. |
| Medical History | Detailed medical history |
| Screen | Records clinically determined demographic data (K-L grade, vital signs, BMI, etc.), as well the findings from the detailed knee exam, which includes palpation for abnormalities, skin assessment, varus/valgus moment, swelling, patellar compression test, and alignment, among others. |
| Med Tracking Log | For recording concomitant medication tracking. |
| Off Study Form | Captures data on when and why patients went off study. |
| Randomization | Ensures all data has been captured prior to randomization, including assurance that last NSAID use was ≥ 1 week and changes to medications. |
| Pre-Treatment Assessment | Includes vital sign data and knee exam findings. |
| Treatment | For recording data on the administration/harvesting for synovial fluid and treatments. Includes, procedure/treatment started/completed times and success/failure data. |
| Post-Treatment Assessment | Repeat of pre-treatment exam. |
| AE Tracking Log | Logs the event description/diagnosis, whether the AE was reported to the IRB, start/end date, action taken, and whether the event was serious or unexpected. |
| Dev Tracking Log | Records deviation information, including determination of whether the deviation impacts safety, data Integrity, or the participant's willingness to participate, as well as whether the event was reported to the IRB. |
| Wound evaluation | *Treatment group only* - Includes vital signs, recording of adverse events/changes to medications, and knee exam findings. Also includes findings from the evaluation of the harvest site, including assessment of the central nervous system, cardiovascular system, allergic reactions, swelling, bruising, fluid drainage, pain or discomfort, and neurologic reactions, among others. |
| Follow-up | Includes recording for adverse events, changes to medications, outcome of synovial fluid sampling, and data from the knee exam and wound evaluation exam |
