# Supplementary material for: A randomized, controlled study to evaluate the efficacy of intra-articular, autologous adipose tissue injections for the treatment of mild-to-moderate knee osteoarthritis compared to hyaluronic acid: a study protocol
Source: BMC Musculoskelet Disord. 2018 Oct 24;19:383. doi: 10.1186/s12891-018-2300-7 (PMC6201482; doi:10.1186/s12891-018-2300-7)
Supplement: Supplementary file 3 — Table S2. Source document schedule. (DOCX 13 kb) [file 12891_2018_2300_MOESM3_ESM.docx]

**Additional File 4**: Supplementary Table 2: Source document schedule

| **Visit** | **Source Documentation Forms to be Completed at Each Visit** |
| --- | --- |
| **Screening Visit** | Eligibility |
|  | Informed Consent |
|  | Screen |
|  | Med tracking log |
|  | Update the following as necessary: Off study |
| **Day 0 – Randomization and treatment** | Randomization |
|  | Pre-treatment assessment |
|  | Treatment |
|  | Post-Treatment assessment |
|  | Update the following as necessary: Med tracking log, Dev tracking log, Off study form |
| **Day 7 - Wound evaluation visit** | Wound evaluation |
|  | Update the following as necessary: AE tracking log, Med tracking log, Dev tracking log, Off study form |
| **Week 6 - Follow-up** | Follow-up |
|  | Update the following as necessary: AE tracking log, Med tracking log, Dev tracking log, Off study form |
| **Month 6 - Follow-up** | Follow-up |
|  | Off_study |
|  | Update the following as necessary: AE tracking log, Med tracking log, Dev tracking log, Off study form |
| **Unscheduled visit** | Follow-up |
|  | Update the following as necessary: AE tracking log, Med tracking log, Dev tracking log, Off study form |
