# Supplementary material for: A randomized, controlled study to evaluate the efficacy of intra-articular, autologous adipose tissue injections for the treatment of mild-to-moderate knee osteoarthritis compared to hyaluronic acid: a study protocol
Source: BMC Musculoskelet Disord. 2018 Oct 24;19:383. doi: 10.1186/s12891-018-2300-7 (PMC6201482; doi:10.1186/s12891-018-2300-7)
Supplement: Supplementary file 4 — ICF document. (PDF 657 kb) [file 12891_2018_2300_MOESM4_ESM.pdf]

Keck Hospital of USC Department of Orthopaedic Surgery
1520 San Pablo St #2000, Los Angeles, CA 90033

Study Title: *A Randomized, Controlled Study to Evaluate the Efficacy of Intra-articular, Autologous Adipose Tissue Injections for the Treatment of Mild-to-Moderate Osteoarthritis*

Principal Investigator: C.T. Vangsness Jr., MD

Study ID: HS-17-00365 Valid From: 6/11/2018 To: 1/27/2019

EXPERIMENTAL SUBJECT'S BILL OF RIGHTS

You have been asked to participate as a subject in a medical experiment. Before you decide whether you want to participate in the experimental procedure, you have a right to the following information:

CALIFORNIA LAW REQUIRES THAT YOU MUST BE INFORMED ABOUT:

1. The nature and purpose of the study.
2. The procedures in the study and any drug or device to be used.
3. Discomforts and risks reasonably to be expected from the study.
4. Benefits reasonably to be expected from the study.
5. Alternative procedures, drugs, or devices that might be helpful and their risks and benefits.
6. Availability of medical treatment should complications occur.
7. The opportunity to ask questions about the study or the procedure.
8. The ability to withdraw from the study at any time and discontinue participation without affecting your future care at this institution.
9. Be given a copy of the signed and dated written consent form for the study.
10. The opportunity to consent freely to the study without the use of coercion.

I have carefully read the information contained above and I understand fully my rights as a potential subject in this study.

Date: ____________________ Time: _________________

Signature: ____

(Research Participant)

INFORMED CONSENT

TITLE: A Randomized, Controlled Study to Evaluate the Efficacy of Intra-articular, Autologous Adipose Tissue Injections for the Treatment of Mild-to-Moderate Osteoarthritis

PRINCIPAL INVESTIGATOR: C. Thomas Vangsness J.r., MD

DEPARTMENT: Orthopaedic Surgery

24-HOUR TELEPHONE NUMBER: 800-872-2273 or 310-704-2269

Study ID: HS-17-00365 Valid From: 6/11/2018 To: 1/27/2019

We invite you to take part in a research study. Please take as much time as you need to read the consent form. You may want to discuss it with your family, friends, or your personal doctor. If you find any of the language difficult to understand, please ask questions. If you decide to participate, you will be asked to sign this form.

This research study is sponsored by the L.K. Whittier Foundation. They provide funding to help cover the costs of this study. The kits for fat harvesting and processing were donated to the study by the manufacturer. Additional funding is provided by the investigator's discretional research account.

WHY IS THIS STUDY BEING DONE?

This study is about fat injections into the knee for people with osteoarthritis. The effects of injected fat treatment for orthopedic use are not well understood. We hope to learn if these fat injections reduce pain and improve knee function compared to hyaluronic acid injections. Hyaluronic acid is the standard of care for pain associated with osteoarthritis. We also hope to understand how these injections effect your knee biology.

You are invited as a possible participant because you are experiencing pain from

osteoarthritis in your knee. About 54 people will take part in the study.

WHAT IS INVOLVED IN THE STUDY?

This study will last 6 months. We ask that you do not take ibuprofen or other similar drugs for the duration of the study. Your knee will be injected one time with either hyaluronic acid (Synvisc One®) or with fat from your abdomen. Both you and the doctor will know what study treatment you are receiving. However, **neither you nor the study doctor will decide which study treatment you will receive**. This determination is made randomly (like flipping a coin). Half of all participants will receive a fat injection. However, if you do not receive the fat treatment and you complete all scheduled follow-up visits, you will be offered the fat treatment at your last follow-up visit free of charge.

Before you receive the study treatment, we will check to make sure you meet the requirements to participate. This is process is called "screening". The screening visit will take about one and a half hours. The following will occur doing your screening visit:

- We will ask you questions about your medical history and medications.
- We will have you answer a short (2-3 minute) questionnaire.
- The study doctor will perform a physical exam
- We will ask you to take a pregnancy test (if applicable)
- You will be asked to stop taking NSAID and narcotic pain medications. NSAIDs are drugs like ibuprofen and Celebrex. Narcotic pain medications are drugs like codeine, Vicodin, and OxyContin. You will not be allowed to take these medications for the duration of the study. If you experience unacceptable pain during the study, we ask that you contact the study doctor before taking any medications. If you are unable to reach the study doctor, you may take 500mg acetaminophen (Tylenol). However, we ask that you keep track of any medication use and report it to the study staff or doctor.

If you do not complete all of the screening procedures, you will be asked to come back.

Study ID: HS-17-00365 Valid From: 6/11/2018 To: 1/27/2019

After you complete the screening visit, it may take up to 3 weeks to make sure you qualify. If you do not meet the requirements for the study, the study doctor will explain why and discuss other treatment options with you.

The injection procedure is the same for both treatments. However, the fat injection involves extra steps because fat must be removed from your abdomen. The procedure for each injection is described below:

Fat injection (About 30 minutes)

1. A solution will be injected into the abdomen to numb the area and loosen the fat.
2. A small amount of fat will be removed (2-6 tablespoons)
3. The knee will be numbed and a small amount of joint fluid will be withdrawn from your knee
4. Using the same needle, the processed fat will be injected into your knee

Hyaluronic acid injection (About 5 minutes)

1. The knee will be numbed and a small amount of joint fluid will be withdrawn from your knee
2. Using the same needle, Hyaluronic acid will be injected into your knee

After you receive the injection, you will need to come back to see the doctor several times. The number of times you need to come back depends on which study treatment you receive. All participants will come back 6 weeks and 6 months after receiving the study treatment. These visits will generally take less than an hour. The study doctor will examine your knee and remove a small amount of joint fluid. You will also be asked to answer several short questionnaires about your knee. These questionnaires will take roughly 5 minutes to complete. Additionally, you will be asked to take a balance test, which involves standing on 1 leg for 20 seconds. A total of four trials (two per limb) will be performed. The balance test will take approximately 3 minutes to complete.

Study ID: HS-17-00365 Valid From: 6/11/2018 To: 1/27/2019

If you receive the fat injection treatment, we will ask you to also come back 2-7 days after you are treated. The purpose of this extra visit is to monitor your belly where the fat was removed. You will not have to answer any questionnaires, and no additional joint fluid will be removed from your knee during this visit.

Genetic research may be performed on your fat and joint fluid samples. Results of this genetic research will not be used in your medical care. The results will not be given to you or your personal doctor.

Information about Samples and Data Collected as Part of This Research
This study involves the collection of joint fluid and excess fat tissue. Providing these specimens is not optional. We need to obtain joint fluid and excess fat tissue for research purposes. These samples will help us understand how the treatment affects the biology of your joint. You will not learn the results of research testing. We do not know how to apply these results to your care. How the tissue is obtained and the risks from getting this tissue will be explained to you separately.

We may use cells from your fat tissue or joint fluid to grow more cells.

WHAT ABOUT PREGNANCY?

We do not know if transferring fat to the knee joint will hurt an unborn baby. If you are pregnant or breast feeding, you cannot take part in this study. If you are a woman who could become pregnant, you must have a pregnancy test to make sure you are not pregnant. If you are a women of child bearing potential, you must use birth control while on this study. These are some birth control measures that you can use:

- Hormonal contraceptives (birth control pills, patches, implants, rings, or injections)
- Barrier methods (such as a condom or diaphragm) used with a spermicide
- An intrauterine device (IUD)

Study ID: HS-17-00365 Valid From: 6/11/2018 To: 1/27/2019

- Surgical sterilization (hysterectomy or tubal ligation for women, vasectomy for men).

WHAT ARE THE POSSIBLE RISKS AND DISCOMFORTS?

1. Removal of fat from your belly region. This is done with a syringe. The procedure is called, "lipoasirpation". The risks associated with lipoaspiration are lower than those associated with liposuction. However, patients sometimes experience one of more of the following:

- Temporary swelling or bruising at the harvest site. These symptoms usually resolve go away within several hours, but may last up to 3-5 days.
- Fluid drainage lasting 3 to 5 days

In very rare cases it is possible that you may experience one or more of the following:

- Headache/nausea lasting a few hours to several days
- Reaction to the local anesthetic (lidocaine and epinephrine):
    - Central Nervous System: lightheadedness, nervousness, apprehension, euphoria, confusion, dizziness, drowsiness, tinnitus (ringing in the ears), blurred or double vision, vomiting, sensations of heat, cold or numbness, twitching, tremors, convulsions, unconsciousness, respiratory depression and arrest; the first sign of toxicity may be drowsiness merging into unconsciousness and respiratory arrest.
    - Cardiovascular system: bradycardia (slow heart rate), hypotension (low blood pressure), and cardiovascular collapse, which may lead to cardiac arrest. In addition, tachycardia (fast heart rate, palpitations, and hypertension (high blood pressure) could occur.
    - Allergic reactions: which can be mild skin rash, itching, or swelling of the skin, lips, tongue, throat, difficulty breathing and rarely life-threatening.

Study ID: HS-17-00365 Valid From: 6/11/2018 To: 1/27/2019

- Neurologic reactions: Persistent paresthesias (an abnormal sensation such as tingling, tickling, pricking, numbness or burning) of the lips, tongue, and oral tissues have been reported with the use of lidocaine, with slow, incomplete, or no recovery.

- Infection
- Temporary or permanent and skin Irregularities
- Damage to nerves or blood vessels

2. Injection of fat into the knee. Possible risks and discomforts include:

- Temporary swelling, bruising, soreness, or numbness at the injection site. These symptoms usually resolve go away within several hours.
- Minor buildup of joint fluid that may last up to several weeks
- Joint inflammation that may last up to several weeks
- Allergic reaction to the local anesthetic which can be mild skin rash, itching, or swelling of the skin, lips, tongue, or in the throat and rarely life-threatening

Note: This treatment is regularly given to people by orthopedic surgeons across the county. However, very little clinical data has been published and we do not fully understand how this treatment will affect you. For this reason, there may be other risks that the investigators did not expect. The investigators will watch you to see if you are experiencing any other side effects.

3. Hyaluronic acid (Synvisc One®) **injections:**

- Temporary swelling, bruising, bleeding, soreness, or numbness at the injection site. These symptoms usually go away within several hours.
- Minor buildup of joint fluid that may last up to several weeks
- Joint pain, stiffness, warmth, and heaviness
- Change in the way you walk (gait disturbance)

Study ID: HS-17-00365 Valid From: 6/11/2018 To: 1/27/2019

- Allergic reaction to the local anesthetic which can be mild skin rash, itching, or swelling of the skin, lips, tongue, or in the throat and rarely life-threatening

Synvisc One® post-marketing experience has identify the following rare systemic (whole body) side effects: rash, hives, itching, fever, nausea, headache, dizziness, chills, muscle cramps, paresthesia (tingling, numbness, prickling sensation in hands, arms, legs or feet), peripheral edema (swelling in hands, arms, legs or feet), malaise (general weakness or discomfort), breathing difficulties, flushing, and facial swelling. There have been rare reports of thrombocytopenia (low blood platelet count) which may increase the risk of bleeding.

Precaution: You should avoid any strenuous activities or prolonged (i.e. more than 1 hour) weight-bearing activities such as jogging or tennis within the 48 hours that follow the hyaluronic acid or fat injection procedure.

4. Joint fluid removal: You may have temporary swelling, bruising, soreness, or numbness where the fluid was removed. These symptoms usually resolve within several days. Allergic reaction to the local anesthetic which can be mild skin rash, itching, or swelling of the skin, lips, tongue, or in the throat and rarely life-threatening

6. Breach of confidentiality: There is a small risk that people who are not connected with this study will learn your identity or your personal information.

7. Stopping pain medications: Stopping your normal medications may result in increased pain. If you feel this pain is unacceptable, tell the study doctor. He will prescribe medication.

Study ID: HS-17-00365 Valid From: 6/11/2018 To: 1/27/2019

WILL YOUR INFORMATION BE KEPT PRIVATE?

We will keep your records for this study confidential as far as permitted by law. However, if we are required to do so by law, we will disclose confidential information about you. The University of Southern California's Institutional Review Board (IRB) may review your records. The IRB is a research review board that is made up of professionals and community members who review and monitor research studies to protect the rights and welfare of research participants.

Officials sent by the Food and Drug Administration (FDA) may look at your research records and medical records. Other people who provide medical care or who handle billing and payment at USC may review your research records and medical records, if necessary to conduct the research. We may publish the information from this study in journals or present it at meetings. If we do, we will not use your name.

You will be asked to sign a separate HIPAA Authorization for Research form authorizing the access, use, creation, and disclosure of your health information. In order to protect your personal information, we will give you a special identification number. Your responses to surveys will be kept for at least 5 years after you complete this study.

WHAT ARE THE POSSIBLE BENEFITS OF TAKING PART IN THIS STUDY?

You may or may not receive any direct benefit from taking part in this study. The treatment you will receive is NOT a cure for osteoarthritis. Hyaluronic acid (HA) injections sometimes relieve pain and improve function, but these effects typically only last 6 months. Fat injections may provide additional benefits, but we do not know for sure and how long they will last. Some people have reported improvements, but this may be because they have an expectation that fat injections will be helpful. It is possible that these treatments do not work as well as hyaluronic acid. It is also possible that they do not work at all.

Study ID: HS-17-00365 Valid From: 6/11/2018 To: 1/27/2019

Your participation in this study will help us learn more about fat injections. We will compare how well fat injections work compared to hyaluronic acid. The samples we obtain will also help us understand how fat injections affect the biology of the knee joint. Additionally, we may gain a better understanding of osteoarthritis as a whole. This could help us develop better treatments in the future.

WHAT OTHER OPTIONS ARE THERE?

An alternative is to not take part and continue with your current care. You do not need to participate in this study to receive these treatments. Fat and hyaluronic acid injections are available outside of this study. There are also alternative treatments available. These include pain relievers and corticosteroid injections. Surgery may also be an option. If you want to know more, the study doctor will explain the risks and benefits of these alternative options to you.

ARE THERE ANY PAYMENTS TO YOU FOR TAKING PART IN THE STUDY?

You will not be paid for participating in this study.

Possible Commercial Products

All tissue and fluid samples are important to this research study. The University of Southern California will own your sample. Samples and the data we obtain from them will be used for research purposes. If you withdraw from the study early, you may request to have your samples destroyed. If a commercial product is developed from this research project, the University of Southern California or its designee will own it. You will not profit financially from such a product.

Cells from your body may be used to start a cell line. A cell line is one that will grow in the laboratory. It may be of commercial value. There is no plan for you to receive payment for any commercial products that are developed.

Study ID: HS-17-00365 Valid From: 6/11/2018 To: 1/27/2019

WHAT ARE THE COSTS?

Some tests and procedures are done for your routine health care, and you would receive them even if you were not participating in this study. You and/or your health plan/insurance will be billed for the tests and procedures you need for routine health care while you are in this study. You will be billed in the same way as if you were not in a study. You will be responsible for any copayments and deductibles required by your insurance. Some health plans/insurance companies will not pay these costs for people taking part in studies. Check with your health plan/insurance company to find out what they will pay for. If you have any questions about which tests or procedures will be billed to you and/or your health plan/insurance, ask the study doctor. You are responsible for the visits and tests that would normally be done as part of the normal standard of care, including:

- The cost of today's visit (your screening visit).
- The costs of tests and procedures that are part of the normal standard of care, including your initial x-ray.
- All costs associated with unscheduled visits to see the doctor.
- Medical care for adverse reactions to the treatment.
- Medical care for any treatment that is not part of the study.
- Any additional medical care that you receive after the 6-month follow-up visit.

Some tests and procedures are done only for research purposes. The study will pay for the following tests and procedures:

- The cost of your first visit (day 0)
- The cost of returning to the doctor for your scheduled follow-up visits. These will take place about 6 weeks and 6 months after you receive the treatment. If you receive the fat treatment, you will be asked to come in for an extra follow-up visit about one week after you receive the treatment. You will not have to pay for this extra visit.
- The pregnancy test (if required) at the screening visit.
- The treatment (hyaluronic acid or fat injection).
- The removal and testing of joint fluid at follow-up visits.

Study ID: HS-17-00365 Valid From: 6/11/2018 To: 1/27/2019

WHAT HAPPENS IF YOU GET INJURED OR NEED EMERGENCY CARE?

If you think you have been hurt by taking part in this study, tell the study doctor immediately. If you require treatment because you were injured from participating in this study, treatment will be provided. You and/or your health plan/insurance will be billed for this treatment. The study sponsor will not pay for this treatment.

There are no plans to offer any type of payment for injury. However, by signing this form you have not given up any of your legal rights.

WILL YOU RECEIVE NEW INFORMATION ABOUT THIS STUDY?

During the study, we may learn new things about the risks or benefits of being in the study. If we do, we will share this information with you. You might change your mind about being in the study based on this information.

WHAT ARE YOUR RIGHTS AS A PARTICIPANT, AND WHAT WILL HAPPEN IF YOU DECIDE NOT TO PARTICIPATE?

Your participation in this study is voluntary. Your decision whether or not to take part will not affect your current or future care at this institution. You are not giving up any legal claims or rights. If you do decide to take part in this study, you are free to change your mind and stop being in the study at any time. You will not lose any rights if you decide to stop being in the study. If the withdrawal must be gradual for safety reasons, the study doctor will tell you.

CAN YOU BE REMOVED FROM THE STUDY?

You may be removed from this study without your consent for any of the following reasons:

- You do not follow the study doctor's instructions
- At the discretion of the study doctor
- The study doctor closes the study

If you are removed from the study, the study doctor will discuss other options with you.

WHOM DO YOU CALL IF YOU HAVE QUESTIONS OR CONCERNS?

You may contact C. Thomas Vangsness, Jr., MD at 323-442-5860 with any questions, concerns, or complaints about the research or your participation in this study. If you feel that taking part in this study has hurt you, please contact Dr. Vangsness immediately. If you have questions, concerns, or complaints about the research and are unable to contact Dr. Vangsness, contact the Institutional Review Board (IRB) Office at 323-223-2340 between the hours of 8:00 AM and 4:00 PM, Monday to Friday. (Fax: 323-224-8389 or email at irb@usc.edu).

If you have any questions about your rights as a research participant, or want to talk to someone independent of the research team, you may contact the Institutional Review Board Office at the numbers above or write to the Health Sciences Institutional Review Board at LAC+USC Medical Center, General Hospital Suite 4700, 1200 North State Street, Los Angeles, CA 90033.

You will get a copy of this consent form.

AGREEMENT:

I have read (or someone has read to me) the information provided above. I have been given a chance to ask questions. All my questions have been answered. By signing this form, I am agreeing to take part in this study.

Name of Research Participant	Signature	Date Signed (and Time*)

I have personally explained the research to the participant using non-technical language. I have answered all the participant's questions. I believe that he/she understands the information described in this informed consent and freely consents to participate.

Name of Person Obtaining Informed Consent	Signature	Date Signed (and Time*)

A witness is required when: (1) the participant cannot see, read, write, or physically sign the consent form, or (2) the Short Form method is used to obtain consent. In these situations, the witness must sign and date the consent form. If no witness is needed, leave this signature line blank.

Name of Witness	Signature	Date Signed

Study ID: HS-17-00365 Valid From: 6/11/2018 To: 1/27/2019
